# The Quarterly Journal of Inebriety

**Published:** 1877-04-20

**Authors:** 


THE QUARTERLY JOURNAL OF INEBRIETY.—The Secretary of the American Association for the cure of Inebriates, favors us with a copy of their journal containing many interesting articles relative to the abuse of alcohol and opium, with the transactions of the Association, and other contributions from leading Specialists in this new field. All communications for subscriptions, etc., should be addressed to T. D. Crothers, M. D. Secretary, Binghampton, N. Y.

We observe, in the opening article by Dr. Crothers, that he regards "Inebriety as a cerebro-psychal disorder, beginning obscurely, followed by complex perversions and degenerations, has a distinct duration, mortality and progress, which can be understood proportionately to the accuracy with which the history of each case is studied," etc. We regret we have not space to copy this valuable paper entire. We wish the Association success in its noble work.

